# Flame Retardant Polyamide Fibres: The Challenge of Minimising Flame Retardant Additive Contents with Added Nanoclays

**DOI:** 10.3390/polym8080288

**Published:** 2016-08-09

**Authors:** Richard Horrocks, Ahilan Sitpalan, Chen Zhou, Baljinder K. Kandola

**Affiliations:** 1 Institute for Materials Research and Innovation, University of Bolton, Deane Road, Bolton BL3 5AB, UK; CZ2BEE@bolton.ac.uk (C.Z.); B.Kandola@bolton.ac.uk (B.K.K.); 2 Faculty of Science, Department of Physics, Eastern University Sri Lanka, Chenkaladi 30350, Sri Lanka; sitpalanahilan@yahoo.com

**Keywords:** polyamide 6, aluminium diethyl phosphinate, ammonium sulphamate, dipentaerythritol, nanoclay, tensile properties, flammability, fabrics

## Abstract

This work shows that halogen-free, flame retarded polyamide 6 (PA6), fabrics may be produced in which component fibres still have acceptable tensile properties and low levels (preferably ≤10 wt %) of additives by incorporating a nanoclay along with two types of flame retardant formulations. The latter include (i) aluminium diethyl phosphinate (AlPi) at 10 wt %, known to work principally in the vapour phase and (ii) ammonium sulphamate (AS)/dipentaerythritol (DP) system present at 2.5 and 1 wt % respectively, believed to be condense phase active. The nanoclay chosen is an organically modified montmorillonite clay, Cloisite 25A. The effect of each additive system is analysed in terms of its ability to maximise both filament tensile properties relative to 100% PA6 and flame retardant behaviour of knitted fabrics in a vertical orientation. None of the AlPi-containing formulations achieved self-extinguishability, although the presence of nanoclay promoted lower burning and melt dripping rates. The AS/DP-containing formulations with total flame retardant levels of 5.5 wt % or less showed far superior properties and with nanoclay, showed fabric extinction times ≤ 39 s and reduced melt dripping. The tensile and flammability results, supported by thermogravimetric analysis, have been interpreted in terms of the mechanism of action of each flame retardant/nanoclay type.

## 1. Introduction

The absence of commercially available polyamide 6 (PA6) fibres reflects the typical high melt reactivity of aliphatic polyamides in general and the related poor potential flame retardant additive compatibilities [1]. Furthermore, flame retardants that are commercially acceptable for bulk and engineering polymers including aliphatic nylons such as PA6, incorporate poly(bromostyrene)/antimony trioxide- and melamine cyanurate-based systems, which are unsuitable for fibres because of the high concentrations (>> 10 wt %) required and the associated reduction in fibre tensile properties [1]. It is well known that inclusion (2–5 wt %) of nanoclays (layered silicates) in polymers such as PA6 improves the latter’s fire performance, typically defined as a reduction in the peak heat release rate and increase in char as determined by cone calorimetry [2,3]. When present together with conventional flame retardant additives, synergistic effects have been seen that enables the possibility of reducing the concentrations of flame retardant present in order to achieve a defined level of overall fire resistance [4,5,6]. This has particular significance in the development of new flame retardant fibres where the addition of normally-required, high levels (typically > 15–20 wt %) of flame retardant severely reduces overall tensile and other desirable textile properties.

Of the many reported academic publications [1,5,6,7,8,9,10,11] on the flame retardancy of polyamides using halogen-free, conventional flame retardants in the presence of nanoclays, two particularly new flame retardant combinations look promising [9,10,11]; where the potential for their application in fibres exists without compromising physical, mechanical and flame retardant properties. The first one is the use of metal salts (notably aluminium) of dialkyl phosphinates developed by Clariant who manufacture a special low particle diameter (D50 ~ 2–3µm, D95 < 10 µm) fibre grade, Exolit OP935. This phosphinate may be used alone or combined with melamine polyphosphate, although in bulk polymers total levels of approximately 15 wt % are required for acceptable levels of flame retardancy [1]. To date however, no commercially successful PA6 fibres have been created based on this agent, although our recent work has shown that the addition of up to 5 wt % aluminium diethyl phosphinate, AlPi, produces marginal reductions in burning rates during the vertical fabric strip burn testing of knitted fabrics but does not produce self-extinction [7]. AlPi in polyamides is believed to function mainly as a gas phase flame retardant, although formation of aluminium phosphate promotes some condensed phase activity in terms of acting as a heat barrier [9].

The second combination described by Lewin et al., which comprises of small total concentrations (<5 wt %) of ammonium sulphamate (AS) and dipentaerythritol (DP) have been shown to promote significant improvements in the flame retardancy of PA6, primarily functioning in the condensed phase [10,11]. Bulk PA6 samples yielded limiting oxygen index (LOI) values > 32 vol % and UL-94 test V-0 ratings, although reductions in both these were observed in the presence of an additional functionalised, montmorillonite clay. We recently extended this work [8] to investigate this system with fixed levels of AS (2.5 wt %) and DP(1 wt %) in the presence of a number of different functionalised clays and fumed silica, each present at 1–2 wt % levels, with a view to developing an optimised system that could be the basis of an acceptable flame retardant PA6 fibre. While this work corroborated that of Lewin et al. [10,11], the effect of an added clay in reducing the melt dripping of PA6 was also identified as a factor in its decreasing the flame retarding effect of the PA6-AS-DP system. We also observed that a pristine, unfunctionalised clay as well as fumed silica partly restored the flame retardancy of the AS/DP combination in terms of increased LOI value. While fibres were not extruded in this work, a significant conclusion was that there is a potential for the development of acceptable, flame retardant PA6 fibres subject to the challenge of being able to accommodate the potential thermal instability of PA6-AS-DP formulations observed during normal melt processing temperatures within the range 260–270 °C for PA6 [12]. More recent work by Coquelle et al. [13] has shown that when up to 5 wt % AS alone is present, PA6 filaments were extrudable to yield undrawn fibres, having tensile strengths of 0.060–0.080 GPa (~7–9 cN/tex) and breaking elongations of about 250%, not much different to those respectively obtained for pure, undrawn PA6 filaments, suggesting minimal degradation. Above 5 wt % AS, filaments became brittle. Unfortunately, insufficient fibres were obtained for conversion into fabric and so no measure of flame retardancy was determined.

There is evidence in many of the previously cited references to suggest that optimal fire performance occurs when the dispersion of both clay and flame retardant are maximised, although the assumed superior performance of nanodispersed relative to microdispersed species is not always clear cut [14]. To improve dispersion, the various strategies used such as multiple-pass compounding [15], use of master-batches [16], use of ultrasound [7,17,18] etc., have shown to improve the overall polymer/fibre properties, including fire performance. In our earlier study we have demonstrated the effect of applying ultrasound during the compounding of PA6 containing flame retardants without/with nanoclays to maximise their dispersion, which improves the tensile as well as flammability of derived filaments [7]. However, in our subsequent work [8], we demonstrated that nanodispersion of functionalised montmorillionite clays such as Cloisite 25A was achieved during simple compounding.

In this work we build on the previous research discussed above [7,8,9,10,11,13]; to more firmly establish whether the incorporation of Cloisite 25A, together with either aluminium diethyl phosphinate (AlPi) or the ammonium sulphamate/dipentaerythritol system at concentrations in PA6 that will enable filaments to be extruded and drawn and which, after conversion into fabrics, yields acceptable levels of flame retardancy. This clay was selected because we had demonstrated its effectiveness in combination in PA6 with both aluminium diethyl phosphinate and the ammonium sulphamate/dipentaethrythritol in our previous work [7,8] and its slightly superior flame resistance, in combination with the latter as measured by LOI. Both tensile and flammability results, supported by thermogravimetric analysis, are used to interpret their respective mechanisms of action.

## 2. Materials and Methods 

### 2.1. Materials

Polyamide 6 was supplied by Solvay/Rhodia, Lyon, France as Technyl C 301 Natural, where “C” denotes the polymer is PA6, the figure “3” denotes medium to high melt viscosity and “0” indicates that it is un-nucleated. Two different, nominally identical batches of this polymer type were used; the first for the PA6/AlPi/clay formulations; the second for the PA6/AS/DP/clay formulations. The respective control PA6 plaque and filaments derived from these batches are denoted as PA6a and PA6b. Flame retardants selected were aluminium diethyl phosphinate (AlPi), Exolit OP 935 (Clariant, Muttenz, Switzerland) (note that this is fibre grade in that its average particle diameter is < 10 µm), ammonium sulphamate (Sigma-Aldrich, Dorset, UK) and dipentaerythritol (Fisher Scientific, Loughborough, UK) and the nanoclay used was Cloisite 25A (a montmorillonite clay functionalised with dimethyl, n-hexyl, hydrogenated tallow quaternary ammonium sulphate) (Rockwood additives Ltd., Southern Clay Products Inc., Louisville, KY, USA). Previous studies using X-ray diffraction [8] showed that this clay was nanodispersed in PA6 following a simple melt processing in a laboratory compounder.

The sample matrix is shown in Table 1 and was designed based on previous experience [7,8] with only one level of clay (2 wt %) being chosen together with either 10 wt % AlPi [7] or 2.5 wt % AS and 1 wt % DP [8] to yield maximum total additive concentrations of 12.0 wt % and 5.5 wt % respectively. Table 1 also lists the various forms of compounded samples (filament yarns, plaques or fabrics) and the test methods by which they were assessed.

Samples were compounded in a Prism Eurolab Compounder having a 16 mm screw diameter and L/D = 24 as described previously with the temperatures along the six zones of the compounder barrel being 210, 225, 230, 230, 240 and 245 °C [7,8]. Extruded polymer water-cooled strands (~2 mm diameter) were collected and granulated into chips for subsequent extrusion into filaments.

For UL94 tests, plaques (ca. 2.5 mm thickness) were prepared from the pellets by compression moulding with spacer plates between aluminium foil-coated steel plates at a set plate temperature of 190 °C for 2.5 min followed by rapid cooling. These were then cut into 130 × 10 × 2.5 mm^3^ specimens.

### 2.2. Melt Extrusion into Filaments

The above compounded samples, dried previously at 80 °C for 24 h, were each extruded into filaments by using a Fibre Extrusion Technology (FET, Leeds, UK) extruder. The filament spinnerette had 20 holes each with a diameter = 0.8 mm. The extruder screw (internal diameter = 21 mm with L/D = 30:1; screw speed 10 rpm) comprised of four different temperature zones and a metering pump/spinnerette zone at 235, 245, 245, and 245 and 245 °C respectively, below the normal 260–270 °C value used for commercial PA6 extrusion [12] to minimise any possible degradation occurring when either AlPi or AS/DP retardants are present.

Extruded filaments were air-quenched (15 °C and 20 m^3^/s flow rate), treated with a proprietary spin finish (JKI-N815, Joongil Oil Chemical Co. Ltd., Jillye-myeon, South Korea) comprising of triglyceride, non-ionic emulsifiers and antistatic agents and passed over three sets of godets to partially draw the filaments. The first set of godets had a surface speed of 100 m/min at 25 °C, the second pair operated at 105 m/min and 50 °C and final set at 130 m/min and 50 °C. Finally, the partly orientated or drawn filaments were wound, using a Leesona winder operating at 135 m/min surface speed thus yielding an overall nominal draw ratio of about 1.3:1. These conditions gave optimum conditions for all filament yarn samples yielding a nominal yarn linear density of 80 tex with an absence of a spinnerette hole blockage and acceptable spinning and drawing performance, in terms of minimal filament breakage and acceptable tensile properties (see Section 3.1). Higher draw ratios were not possible because of excessive filament breaks at this velocity and lower velocities were not possible arising from excessive molten filament stabilities, a consequence of the relatively low, apparent molten polymer viscosities.

### 2.3. Fabric Production

For each filament, partly drawn yarn sample, sufficient lengths were extruded to knit them into fabrics using a hand-powered V-bed rib flat machine operating with 11 gauge needles. The area density of each fabric was controlled by the gauge of the machine and covered a range 400–509 gm^−2^ in spite of efforts to control values close to a nominal value of 450 gm^−2^ for PA6 comprising AlPi and/or Cloisite and in the range 527–638 gm^−2^ for the PA6 fabric set comprising AS, DP and/or Cloisite 25A. The final differences between fabric values reflected the respective filament yarn linear densities and fabric stitch densities.

### 2.4. Tensile Properties

Tensile testing of extruded, partly drawn filament yarns was carried out using a Textechnic Statimat M Tester (Textechno, Moenchengladbach, Germany) with a gauge length of 100 mm, load cell 10 N and test speed of 300 mm/min (to BS1932:2:1989). At least ten different partly orientated yarns from the same sample were tested, the results averaged and coefficients of variability calculated for respective, average initial Young’s modulus, tenacity and elongation-at-break values.

Additional tensile testing of the partly orientated PA6/AS/DP-containing set of yarns was conducted using an Instron tensile tester (Instron Ltd., High Wycombe, UK) with a gauge length of 100 mm, load cell 10 N and test speed 100 mm/min to initially elongate them by an additional 100% equivalent to a draw ratio of 2:1 and respective sample linear densities determined. This drawing and subsequent testing was carried out for this set of yarns only because of concerns of the effects that excessive thermal degradation might have based on the observations of Coquelle et al. [13]. These yarn filaments were considered to be fully drawn (with a total nominal draw ratio = 2.3:1) and hence highly orientated. Full tensile testing of these drawn filaments occurred under the conditions used for partly orientated yarns and the values of initial Young’s modulus, tenacity and elongation-at-break averaged as above.

### 2.5. Flammability Testing

Limiting oxygen index values were determined for cast plaques, prepared by hot pressing at 190 °C for 3.5 min according to ASTM D2863 with sample sizes 100 × 10 × 3 mm^3^.

UL-94 testing in both horizontal and vertical geometries was carried out on hot-pressed bar specimens of each sample each with dimensions of 130 × 10 × 2.5 mm^3^ cut from cast plaques. Five replicas of each sample for UL-94 vertical and two replicas of each sample for UL-94 horizontal testing were used.

Vertical flame spread tests on knitted fabric samples were undertaken using the previously described method [16], which was based on a modification of the BS 5438:1976: Test 1 and BS5438:1989 [19] used for vertical ignition testing of fabric samples 190 × 70 mm^2^ in size. Samples of this same size were marked at 10, 60, 120 and 180 mm intervals. The first 10 mm of sample burning was not taken into account and so times of burning were recorded from this point onwards after the standard flame had been applied for 10 s to the bottom edge of the fabric, as specified in BS 5438:1989. Video recordings of the burning of each sample were undertaken, times to reach first the 60 mm and, if still burning, the 110 mm marks (=100 mm burnt length) and/or to achieve flame extinction were noted. Where possible, the average number of drips was counted during the entire burning period for each sample enabling average drip rates to be calculated. Three replicates of each sample were burnt and results averaged. The burning behaviour of each sample was observed and recorded digitally.

### 2.6. Thermal Analysis

Thermogravimetric (TGA) and differential thermal analysis (DTA) were undertaken for both control PA6 samples and TGA for all compounded samples in Table 1 as granules using a TA System STD 2690 (TA Instruments Ltd., Elstree, UK) combined TGA/DTA instrument under both nitrogen and air atmospheres (100 mL/min) at a heating rate of 10 °C/min and a sample size of 8–10 mg.

## 3. Results and Discussion

In order to simplify sample notations, the PA6 “a” and “b” suffixes will be omitted in the tables and discussion below since all AlPi/25A samples comprise PA6a and AS/DP/25A samples contain PA6b polymer (see Table 1).

### 3.1. Tensile Properties

#### 3.1.1. Partly Drawn Yarns

In order to assess whether the yarn properties have been influenced by either the additives present and/or accompanying thermal degradation during extrusion, the results in Table 2 are expressed as relative values to those for initial modulus, tenacity and percentage elongation-at-break for the respective partly drawn PA6 yarns for which actual values are given in brackets. As stated in Section 2.1 both sets of yarn samples were prepared at different times (with different batches of PA6) and processing parameters had to be adjusted based on respective additives used. Hence yarn tensile properties of the respective control PA6 samples were different for both sets of samples; results for both sets are given in Table 2 and for flame retarded yarns, the values are presented as relative values. Possible reasons for these differences will be discussed below when the thermal and flammability characteristics are discussed. The average coefficient of variation across the PA6/AlPi data sets is 11% while those for the PA6/AS/DP yarns is 17%. The higher value for the latter reflects the greater probable level of degradation present (see below). In Table 2 are listed also the multifilament yarn linear densities which for the PA6/AlPi yarn set are within the range 82–87 tex and for the PA6/AS/DP set a higher, but consistent value of 101 tex, which given the possible rheological differences caused by additive presence and possible degradation, are still quite close to the nominal 80 tex value.

Generally it has been reported that a well-dispersed nanoclay increases the initial modulus, reduces breaking elongation and has a small effect on tenacity of filaments [4]. For the PA6/AlPi yarn set, within the stated error, it is clear that very little changes in initial moduli are observed for all formulations relative to the 100% PA6 yarn, with only the PA6/AlPi(10%)/25A(2%) yarn showing a significant increase. Tenacity values are more variable with a considerable decrease observed for the PA6/AlPi(10%) yarn suggesting that both the presence of the additive as well as possible accompanying degradation maybe responsible. The further addition of 2 wt % clay appears to partly offset this effect. Breaking elongations or strains are usually susceptible to both the presence of an additive acting as a filler as well as any accompanying degradation. Here it would seem again that the presence of AlPi alone has a significant reducing effect on this parameter, offset by the further addition of clay, which parallels the reduced, respective tenacity values. In summary therefore, it would appear that while the introduction of aluminium diethyl phosphinate alone promotes some reduction in tensile properties, the initial modulus is largely unchanged and the further addition of clay partly reduced these reductions.

For the PA6/AS/DP yarn set, while the extrusion machine variables were set to yield a nominal linear density of 80 tex, slight changes relative to the actual control PA6 yarn value (77 tex) were made following small windup rate variations undertaken to ensure optimum filament stability during extrusion; hence the variations in yarn linear density reported in Table 2. These were necessary because the presence of additives influenced the melt viscosity most likely as a result of thermal degradation, which is most likely reflected in the higher yarn property, average coefficients of variation of 17%.

In spite of the increased error, surprisingly, given the known thermal degradative sensitivity [10,11], the initial Young’s modulus increases with the addition of the AS/DP combination and further increases in the presence of the 25A clay. Respective sample tenacities however, show consistent decreases relative to the respective PA6 yarn, although the initial large decrease when AS/DP alone is present, is partly restored on the further addition of clay and as observed, also for the PA6/AlPi yarn set.

Percentage breaking elongations however, also show the surprising suggestion of an increase upon the addition of AS(2.5%)/DP(1%), with a more significant increase occurring when clay is also present. This is the opposite effect seen in the PA6/AlPi/25A formulations, where both tenacities and breaking elongations are reduced relative to 100% PA6. If the average molecular weight of a polyamide polymer is significantly reduced as a consequence of degradation, this is usually accompanied by a decrease in elongation-at-break and not the converse as seen in Table 2. Thus the general increase in initial Young’s moduli and breaking elongations would suggest that in spite of its sensitivity to thermally degrade, the AS/DP combination has promoted little chain scission, although this conclusion is partly offset by the reductions in tenacity.

#### 3.1.2. Fully Drawn Yarns

As discussed above however, Coquelle et al. [13] rule out any significant degradation in terms of loss in tenacity when PA6 containing 5 wt % AS only was extruded (also at 245 °C), to yield undrawn filament tenacities in the range of ~7–9 cN/dtex. In order to be able to assess more accurately the effects of thermal degradation, each partly drawn PA6/AS/DP sample was cold drawn using the Instron tensiometer with a draw ratio of 2:1 yielding a total draw ratio of 2.6:1, including the 1.3:1 ratio introduced during the extrusion stage. Table 3 shows that the fully drawn yarns have linear densities reduced by about 50% as expected and values of modulus and tenacity which have increased significantly when compared with respective undrawn values in Table 2.

The respective tensile properties for 100% PA6 drawn yarns compare well with typically quoted commercial modulus and tenacity values of 100–300 and 25–35 cN/tex values respectively. The breaking elongation values have reduced by almost an order of magnitude compared with their respective, partially drawn analogue yarns as expected and again are similar to commercial values usually in the range 25%–40%. The same trends in terms of increased moduli and breaking elongations following the introduction of 2.5 AS, 1 wt % DP and subsequently 2 wt % clay observed in Table 2 for partly drawn yarns are reflected for the fully drawn yarns in Table 3. Closer inspection of both Table 2 and Table 3 with regard to tenacity shows that in spite of the associated error, tenacity values for PA6/AS/DP-containing yarns are consistently but slightly enhanced when clay is added, suggesting that the latter is having a matrix-reinforcing character in spite of any thermal degradation present [20].

### 3.2. Flammability

Because fabric area densities and component yarn linear densities varied across the samples to be studied and these factors are known to influence LOI values, for example [21], LOI and UL-94 tests were performed on cast plaques, both of which had gone through a second thermal processing, similar to the one for yarn production. Thus the respective formulation burning behaviours could be compared free of physical structural variations.

#### 3.2.1. Limiting Oxygen Index of Plaques

Limiting oxygen index values for both sets of plaques together with respective PA6 control samples are listed in Table 4. It is noteworthy that both control PA6 samples have quite different LOI values which, like the observed differences in respective tensile property differences, we can only ascribe to the fact that two different batches having slightly different chemical characteristics but of the same nominal type of commercial polymer were used, as stated above. With respect to the PA6a control sample, the presence of 2% Cloisite slightly reduces the LOI value and this effect has been noted previously and ascribed to the reduced melt dripping, which prevents the flaming drips from leaving the flame zone [4]. The presence of 10 wt % AlPi alone increases the value considerably, which on further addition of 2 wt % Cloisite 25A raises the LOI to 29.0 vol %. In our earlier work on PA6/AlPi/25A formulations where LOI values of extruded strands of about 1.2 mm diameter were determined, a similar decrease in LOI value to 18.8 vol % was observed following the addition of 2 wt % Cloisite 25A; and with the addition of 5 wt %AlPi, the LOI increased only to 21.1 vol %. However, it is well known that flame retardant properties are not linearly related to flame retardant content and the recommended concentration of AlPi to guarantee a high UL94 V-rating is at least 15–18 wt % [1]. In earlier work we showed that 7.5 and 10 wt % AlPi levels in PA6 produced respective LOI values of 23.9 and 24.8 vol % and UL94 “fails” when tested vertically [22]. While the value for 10% AlPi level in Table 4 is higher than the latter, there is consistency between the UL94 results (Table 5).

The LOI results for the second set of formulations shows that the addition of 2.5%AS and 1%DP raises the LOI to 27.7 vol % and addition of clay reduces this slightly to 24.9 vol %. This reduction of 2.8 vol % corroborates the previous observations of Lewin et al. [10,11] who attributed a similarly observed decrease in flame retardancy to the large surface area of the exfoliated clay adsorbing and thus deactivating a part of the combined AS and DP flame retardant.

It is therefore apparent that in terms of LOI, the flame retarding effect of 10 wt % AlPi is similar to that of 2.5 wt % AS and 1 wt % DP but the further addition of Cloisite 2A, while promoting increased retardancy in the former, antagonizes that of the latter.

#### 3.2.2. UL94 Results of Plaques

Table 5 lists the results of the horizontal and vertical orientated plaque samples for PA6/AlPi and PA6/AS/DP formulations. Times for the flame to extinguish after first (*t*_1_) and second (*t*_2_) ignitions were recorded and averaged to the nearest second. The melting/flaming drips were counted by using the video clips for the corresponding experiments and dripping rates (DR) during the first ignition are included in Table 5. According to the vertical UL-94 test result from the point of view of the pass/fail criteria, all PA6/AlPi formulations failed while the PA6/AS/DP samples passed with a V-2 rating. That higher (e.g., V-0) ratings were not achieved was disappointing and in the case of the PA6/AlPi/25A formulations, unexpected, given the high LOI value shown in Table 4. Once again, these observations demonstrate the absence of any simple relationship between LOI values and UL-94 ratings.

The presence of clay alone in sample PA6/25A(2%) increased the burning time of PA6, because nanoclay consolidates the molten polymer mass and decreases dripping. For PA6/AlPi samples tested vertically, the presence of flame retardant alone, or together with the clay, helped to reduce the burning rate significantly as did the tendency to form molten drips; and DR reduced to almost zero. However, no V-ratings were achieved for samples containing AlPi in spite of the relatively high LOI values recorded in Table 4, as noted above. In the horizontal mode, the PA6/AlPi(10%) clearly was the most flame retardant with *t_1_*, *t_2_* = 0 s and the effect of added clay was to increase the tendency to remain ignited after withdrawal of the igniting flame. The additional presence of clay increased burning times in both geometries while hardly affecting the reduced dripping rates caused by AlPi.

However, in PA6/AS/DP samples the flammability was significantly reduced and in spite of the dripping being largely unaffected and a lower LOI value than was achieved by the PA6/AlPi/25A set of samples (Table 4), a V-2 UL94 rating was achieved. Closer inspection of the PA6/AS/DP results suggest that in the horizontal mode, the presence, or absence of clay has little noticeable effect, although the vertical UL94 results suggest that the added presence of Cloisite 25A increases *t*_1_ and *t*_2_ slightly, thus mirroring the reduction in LOI shown in Table 4. In both vertical and horizontal orientations, the presence of clay increased the dripping rate.

#### 3.2.3. Vertical Strip Testing of Fabrics

**Figure 1:**
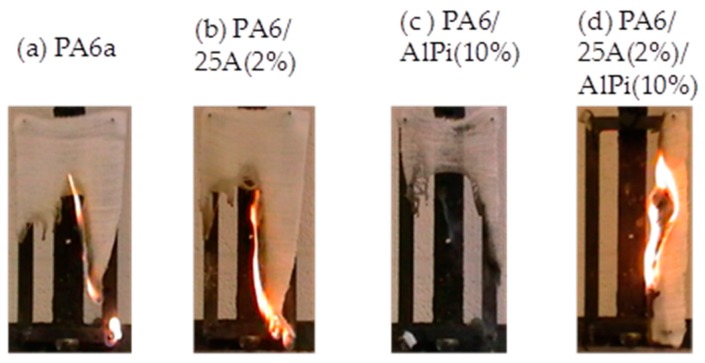
Selected images of burning PA6 fabrics comprising AlPi and/or Cloisite 25A 40 s after extinction of the igniting flame.

**Figure 2:**
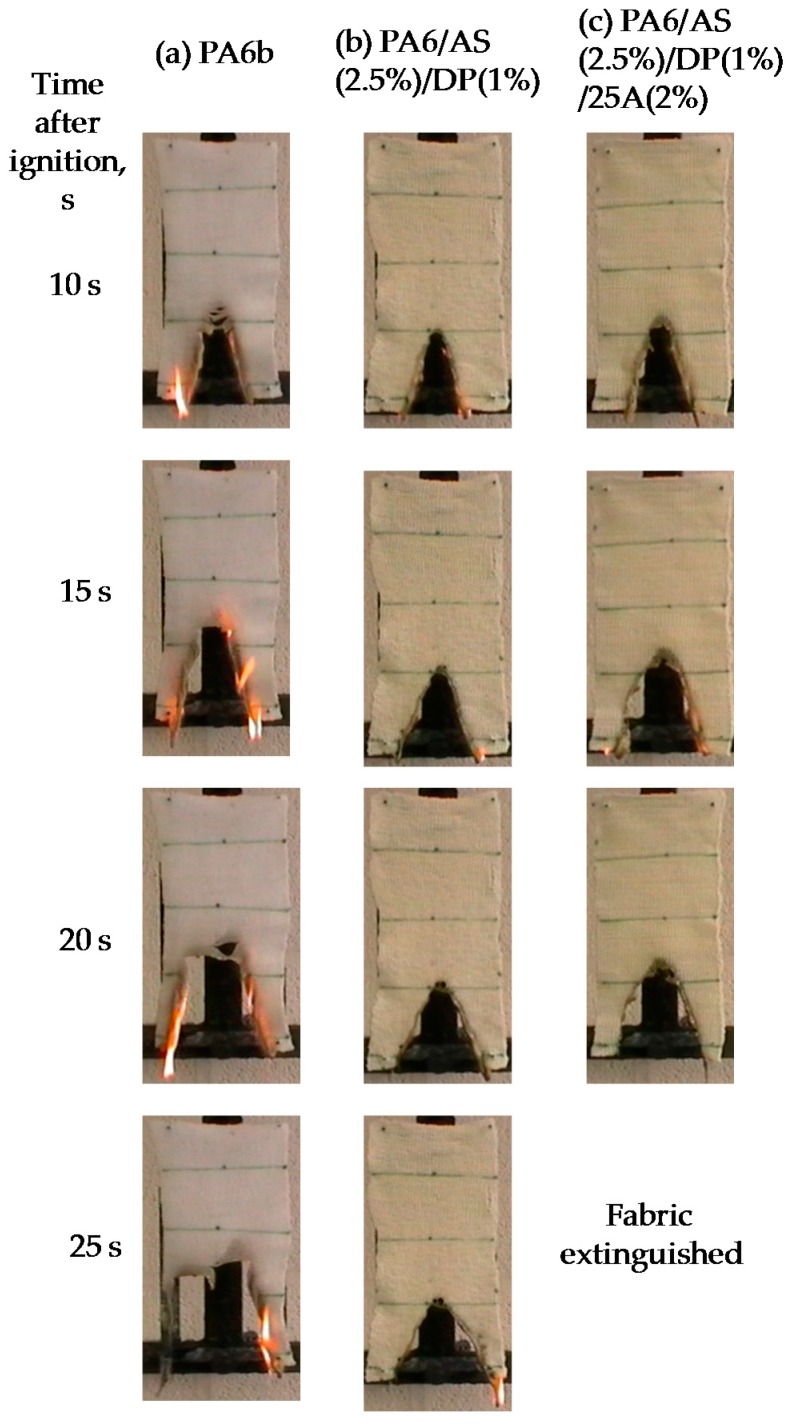
Burning and extinguishing behaviour of ignited PA6/DP/AS/clay-based fabric samples: 10, 15, 20 and 25 s after extinction of the igniting flame.

All filaments were knitted into fabrics and flame spread tests were undertaken using conditions as described in Section 2.5, using the modified BS 5438 method. These vertical strip burning results are listed in Table 6 and selected images of burning fabrics are shown in Figure 1 for PA6/AlPi samples and in Figure 2 for PA6/AS/DP samples.

Table 6 also lists the respective knitted fabric area densities which range from 400 to 505 gm^−2^ in PA6/AlPi samples and 542 to 638 gm^−2^ in PA6/AS/DP samples. Since not all fabrics burnt the entire length (see Figure 1 and Figure 2), respective fabric burnt lengths, time to burn that length (or extinguishment time), rate of flame spread and the average number of molten drops were recorded for each sample in triplicate.

Of the PA6/AlPi/25A fabric sample set, only the PA6/AlPi(10%) sample showed any tendency to self-extinguish, although its burnt length was the same as that of the control fabric. This is also shown in Figure 1, which presents selected sample burning images, each recorded at 40 s after the extinction of the igniting flame. From Table 6, it is clear that the PA6/AlPi(10%) sample, while showing self-extinguishing properties, also produced the highest flame spread or burning rate after sample ignition as well as a much lower tendency to drip. On further addition of 2 wt % Cloisite 2A, the burning rate reduced, the sample was not self-extinguishing and the dripping tendency reduced further. The trend is similar to that observed in the UL94 test. However, it may be concluded that there is no simple correlation between self-extinction and burning and drip rates, although the first property is one that would be associated with an acceptable level of flame retardancy in a standard vertical strip test. Close inspection of Figure 1c of the extinguished residue of the PA6/AlPi(10%) fabric suggests some small level of char formation which is corroborated by the TGA evidenced in Table 7 below. However, similar char formation is not seen in the PA6/AlPi(10%)/25A(2%) fabric residue because it did not self-extinguish.

In PA6/AS/DP fabric samples all fabrics, including 100%PA6 self-extinguished within a time of ≤51 s with the PA6/AS(2.5%)/DP(1%) and PA6/AS(2.5%)/DP(1%)/25A(2%) samples at ≤31 s. The reduced burning time and burnt lengths of this control PA6 fabric, relative to that for the PA6/AlPi samples, is a consequence of the higher area density of the former. Presence of the AS and DP flame retardant components only decreases the burning time by about 40%, the burnt length by about 40% and the drip rate by about 64% with respect to pure PA6 and these parameters are further reduced with the addition of Cloisite 25A organoclay. The reduction in drip rate, while being significant when AS and DP alone are present, is not as great as seen for the PA6/AlPi(10%) fabric, although a similar reduction occurs again with the further addition of Cloisite 25A. A comparison of Table 5 and Table 6 however, show that the changes in drip rate in PA6/AS/DP/clay fabrics are not reflected in the UL94 testing of plaques. Whether for this same set of fabrics, the reduced dripping tendency is paralleled by a char increase is not clear from the burning strip images in Figure 2. However, the TGA data under air conditions shown below in Table 7 gives an almost zero mass residue at 600 °C for the PA6/AS(2.5%)/DP(1%) sample. This may be compared with a residue of 2.0% for the PA6/AS(2.5%)/DP(1%)/25A(2%) sample, which if the clay content is taken into account, represents a negligible, additional carbonaceous content.

Figure 2 illustrates how the flame spread behaves during the flame spread test for the first 25 s after extinction of the igniting flame and these clearly show the superior self-extinguishing behaviour of the PA6/AS/DP/25A sample. The LOI values listed in Table 4 for plaques, however, do not appear to correlate well with the general order of increasing flame retardancy determined by fabric burning times and lengths:

PA6/AS/DP/25A > PA6/AS/DP >> PA6

While the decreasing LOI order is:

PA6/AS/DP > PA6/AS/DP/25A > PA6

In conclusion, it is clear that the PA6/AS/DP/clay-containing fabrics show definite self-extinguishing characteristics unlike the PA6/AlPi/clay-containing series.

### 3.3. Thermogravimetric Analysis

**Figure 3:**
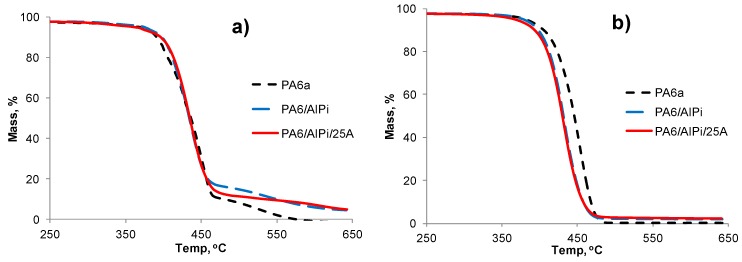
PA6/AlPi/clay samples in (**a**) air and (**b**) nitrogen.

**Figure 4:**
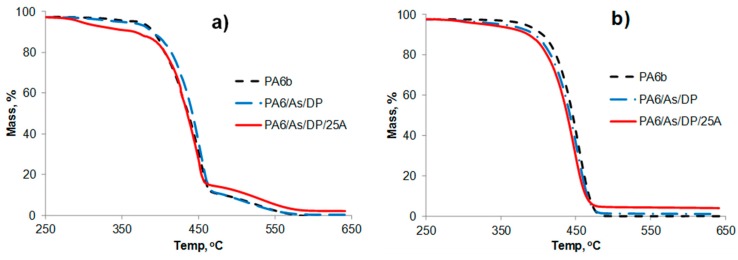
PA6/AS/DP/clay samples in (**a**) air and (**b**) nitrogen.

**Figure 5:**
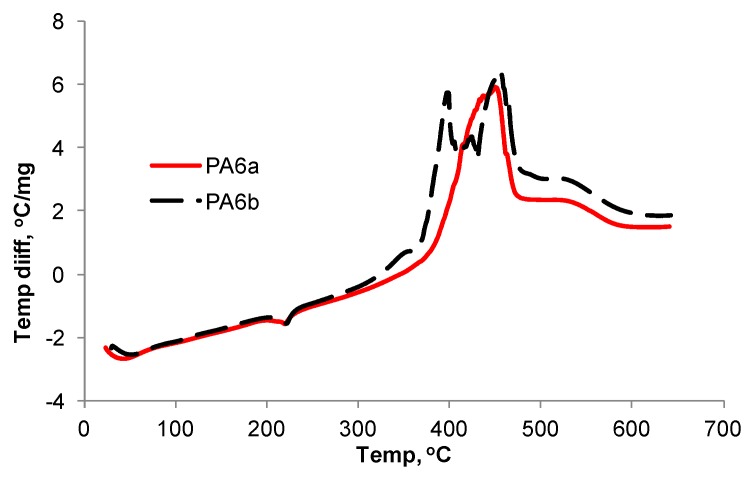
DTA responses in air of PA6a and PA6b control samples.

In order to attempt to explain the differences in tensile and LOI behaviour between the two control PA6 samples, the respective TGA and DTA results were compared. The TGA curves for all samples including those under air and nitrogen are shown in Figure 3 and Figure 4. TGA data, expressed in terms of 5% mass loss (*T*_5%_), 10% mass loss (*T*_10%_) and 50% mass loss (*T*_50%_) temperatures, are listed in Table 7 together with respective residue values at 600 °C. The latter under nitrogen would comprise both carbonaceous (char) and inorganic contents while in air, carbonaceous content would have been oxidised. For both controls, the only significant differences are their respective *T*_5%_ values of 364 versus 371 °C under air and 388 versus 382 °C under nitrogen. This suggests that the presence of air sensitises the thermal degradation of PA6a more than PA6b but that the converse is true under nitrogen. However, since the samples were passed through the compounder and extruder in the presence of air, it was considered that both TGA and DTA behaviour in air were worthy of further analysis. Respective DTA curves are shown in Figure 5 from which it is seen that above their respectively similar melting endotherms, a significant difference in their respective thermal behaviours is observed. The PA6a sample is characterised by a single exothermic degradation peak at 446 °C with a slight shoulder at 442 °C, while PA6b exhibits two peaks at 396 and 454 °C. Clearly these two control samples, although of the same nominal grade, differ in their respective thermal behaviour, although respective TGA results in air suggest only a very slight difference to be present. Unfortunately by the time the experimental work had been completed, there were no remaining quantities of each control polymer left for further analysis. The very small, slightly different residues under nitrogen at 600 °C for both samples, while reflecting the generally known absence of an intrinsic char-forming character in PA6 [1], might also relate to their observed thermal behaviours.

The TGA responses and thermal data in both air and nitrogen atmospheres are also shown in Figure 3 and Figure 4, show for the respective PA6/AlPi/clay and PA6/AS/DP/clay formulation sets respectively in air and nitrogen, together with thermal data listed in Table 7.

#### 3.3.1. PA6/AlPi/Clay Formulations

Cursory analysis of Figure 3a,b shows that in air very little change in mass loss trends occurs up to about 450 °C in air for all three samples, whereas in nitrogen the presence of AlPi alone or with clay shifts their TGA response curves to an almost identical lower temperature regime with respect to the PA6 control. A similar shift of responses under nitrogen was recorded by Ramani et al. [6,23] for PA6 containing Exolit 1311, which comprises of AlPi and melamine polyphosphate (MPP) in a mass ratio 2:1 and the clay Cloisite 30B, which is a montmorillonite clay exchanged with methyl octadecyl (tallow) bis-2-hydroxy ammonium ion (III). The presence of either Exolit 1311 at 18% or 1331 with 30B at 5% both caused similar shifts with respect to the PA6 control.

Comparison of the 5%, 10% and 50% mass loss temperatures in Table 7 reflects quantitatively these general observations for our PA6 formulations. Under nitrogen, the observations of Braun et al. [9] suggest that very little interaction between the PA6 and the added aluminium diethyl phosphinate occurs up to about 400 °C, although they report a slight reduction in the onset of degradation. These same workers propose that heating PA6 and AlPi under nitrogen involves the simultaneous volatilisation of AlPi and its decomposition to the also volatile diethyl phosphinic acid; and so the greater mass losses from the samples containing AlPi above, temperature may be partly ascribed to these effects. However Table 7 shows that *T*_5%_ values under nitrogen decrease from 388 °C for the PA6 control to 374 °C in the presence of 2% Cloisite 25A alone, to 379 °C in the presence of 10% AlPi and to 368 °C when both are present. *T*_10%_ values also show respective decreases from 407 to 400, 398 and 394 °C suggesting that both clay and AlPi individually affect the mass loss in this temperature region. *T*_50%_ values suggest that the temperature reducing effect of AlPi is much greater than the clay, further reflecting its probable loss by volatilisation and decomposition to diethyl phosphinic acid [9].

The addition of 10% AlPi increases residual char formation above 450 °C (Figure 3a) although in air (Figure 3b) this has been clearly oxidised leaving only an inorganic residue above this temperature. The char-promoting character of AlPi was noted previously during the fabric flammability tests shown in Figure 1c. There are however, differences in residue levels at 600 °C between respective samples heated under nitrogen and air, where little or no char should be expected to be present. Residues of samples containing only 2% 25A under both nitrogen and air reflect this initial content less the organofunctionalising species initially present. Under nitrogen, the increase in value of 0.9% for the PA6/AlPi residue with respect to the PA6 control will partly reflect the non-volatile aluminium content of the original phosphinate, most likely aluminium phosphate [9]. However, the residue value for the PA6/AlPi(10%)/25A(2%) sample at 600 °C, appears to be slightly less than the sum of the residue values of the PA6/25A(2%) and PA6/AlPi(10%) samples, although the error in measuring very small residue levels is probably significant.

In air, the behaviour is quite different as noted above. Figure 3a shows that only after about 450 °C do the various TGA response curves separate, although the *T*_5%_ values in Table 7 suggesting that the presence of clay slightly destabilises PA6, while AlPi stabilises it to oxidative degradation, results that are also reflected in respective *T*_10%_ values. These results conflict with those of Ramani et al. [23] for PA6/AlPi/MPP/30B combinations in air, in which reported TGA responses in air are similar to those in nitrogen and hence similar to our results in Figure 3b. However, the presence of MPP, which starts to decompose over the range 300–350 °C and should be little affected by the presence of air, is most likely responsible for this apparent insensitivity to the presence of air. Jahromi et al. [24] have also produced evidence that MPP causes depolymerisation in PA6 when heated at 350 °C.

In Table 7, *T*_50%_ temperatures occur for all samples in the range 434–436 °C, which is the point at which the curves in Figure 3a intersect. Clay sensitisation of PA6 in air up to 300 °C was reported to be negligible by Bourbigot el al. [25] and Ramani et al. [23], where the clay used was Cloisite 30B. Above 450 °C the presence of this clay alone promotes a small amount of char formation. As mentioned above, this clay contains two primary hydroxyl groups in the organofunctionalised component, which together with the PA6, appear to decompose independently up to 450 °C. The organofunctional group in Cloisite 25A has methyl groups replacing the primary hydroxyl groups and so would be expected to be less reactive. Furthermore, in the presence of air Samyn and Bourbigot [26] corroborated the earlier work by Braun et al. [9] in that decomposition of AlPi yielded aluminophosphates giving rise to greater residues. This latter oxidative decomposition explains why the TGA responses for PA6 and PA6/AlPi in air are similar up to about 450 °C (Figure 3a) because the decomposition of AlPi to aluminophosphate species will most likely offset the mass loss due to AlPi volatilisation and the formation of the volatile diethyl phosphinate (Figure 3b).

Residues at 600 °C in Table 7 in air show that in the PA6/25A sample, the value reflects the original weight of clay present, while the PA6/AlPi sample has an elevated value of 4.6% due to the presence of aluminium phosphate. The residue for the PA6/AlPi/25A sample will comprise both inorganic components.

#### 3.3.2. PA6/AS/DP/Clay Formulations

Figure 4a shows that under nitrogen the presence of ammonium sulphamate and dipentaerythritol sensitises the degradation of PA6 as expected from the work of Lewin et al. [10,11] and it is further sensitised when Cloisite 25A clay is present. Table 7 shows this effect in terms of the respective significant reductions in *T*_5%_ values. Previous work by Lewin et al. [10,11] proposed sulphation reactions and ammonia release occurring in the temperature range 200–270 °C under nitrogen between PA6, AS and DP. Under air conditions, Figure 4b suggests that there are only slight differences generally although shifts of *T*_5%_ values to lower temperature following the addition of AS and DP and then 25A are increased from 371 to 344 to 295 °C respectively (see Table 7). These shifts are reflected also for *T*_10%_ and *T*_50%_ values under both nitrogen and air conditions. During the normal extrusion of PA6 fibres in the 260–270 °C range [12], it is obvious that the PA6/AS/DP samples alone or in the presence of additional clay will experience some thermal degradation since Lewin et al. [10] proposed that while sulphation of alkylamide chains occur in the 220–275 °C range, only above 275 °C does the AS cause chain scission. Here, more ammonia and water are released as well as AS reacting with amine end groups present in PA6 to give rise to PA6-AS-DP cross-links and hence char formation. They also propose that the presence of an organoclay sensitises these reactions and lowers the higher temperature threshold to about 240 °C [11]. This sensitisation probably explains the differences in reductions in *T*_5%_ values between the PA6/AS/DP and PA6/AS/DP/25A samples, although greater in air then in nitrogen. However, that both *T*_5%_ values in both nitrogen and air are well below 375 °C at which mass loss from evolution of ammonia formed during chain scission reactions may become significant [10] (corroborated by our value *T*_5%_ = 371 °C for the PA6b control in Table 7), suggest that levels of thermal degradation may again be acceptable during melt compounding and extrusion into filaments. In the presence of AS and DP under air atmosphere, Table 7 shows that *T*_10%_ reduces to 389 and then to 366 °C when 2 wt % Cloisite 25A is also present, showing that volatilisation is now closer to and most likely associated with the evolution of ammonia due to chain scission. Residues at 600 °C under nitrogen are increased first by the addition of AS and DP and then again by 2 wt % Cloisite 2A suggesting slight char enhancement in both cases. Under air conditions, following char oxidation, respective values reflect any residual inorganic content (principally the clay).

It should be noted that when extruding the PA6/AS/DP/25A formulations with a final barrel and die temperature of 245 °C, filaments were controllable, appeared to be free of any visible discolouration and enabled facile conversion into partly orientated filament bundles. The tensile property results in Table 2 show a significant reduction in tenacity of partly orientated PA6/AS(2.5%)/DP(1%) filaments with regard to PA6, which is also seen in Table 3 for the respective fully drawn yarns, suggesting that some chain scission and hence reduction in average molecular weight has occurred. Clearly, further work to confirm that this proposal is valid.

## 4. Conclusions 

It is well known that the production of flame retarded PA6 fabrics with self-extinguishing properties in which component fibres still have acceptable tensile properties requires low levels (preferably ≤ 10 wt %) of flame retardants which would need to demonstrate highly effective flame extinguishing properties. This work has compared the flame retarding effectiveness of the recently commercialised aluminium diethyl phosphinate (AlPi) present at 10 wt % with the ammonium sulphamate/dipentaerythritol system reported by Lewin et al. [10,11] present at 2.5 and 1 wt %, respectively. Both systems have been investigated in the presence and absence of the organofunctionalised nanoclay, Cloisite 25A. However, it is evident that there is a slight difference in the chemical character of both control PA6 polymers and derived plaques and yarns which may be responsible for PA6a partly drawn filament yarns having modulus and tenacity values more than twice those for PA6b samples (Table 2) and a greater LOI value (22.2 vol %, Table 4). As stated above however, the difference in respective fabric burning properties is more likely to be a consequence of the difference in area densities. Notwithstanding these differences in the respective control PA6 yarns, within each set of formulations there appears to be consistency of results generally.

In terms of flammability, none of the AlPi-containing fabrics achieved self-extinguishability, although the PA6/AlPi(10%)/25A(2%) sample had a low burning rate and the lowest level of melt dripping. Both AS/DP-containing formulations with total flame retardant levels of 5.5 wt % or less showed self-extinction times of 31s or less and reduced dripping tendencies when tested as fabrics in a vertical orientation. The further addition of Cloisite 25A clay reduced the extinction time to about 23 s which is reflected in the slight increase in LOI from 27.2 to 29.0 vol % (Table 4). These same filaments also show superior tensile properties in terms of elevated initial Young’s modulus relative to 100% PA6 and PA6/AlPi-containing filament yarns. The role of additional clay appears to be inconsistent in the two sample sets in that its presence increased both the burn time and burnt length for the PA6/AlPi(10%)/25A(2%) fabric but reduced both parameters for the PA6/AS(2.5%)/DP(1%)/25A(2%) fabric (Table 6), in spite of the claimed deactivating effect that an organoclay has on AS/DP by Lewin et al. [11] and observed as a reduction in LOI in Table 4. The UL94 vertical orientation results for the former’s parent polymer reflects this effect of added clay in terms of increased average burning time, *t_1_*, but no marked effect is shown for the latter’s polymer plaque *t_1_* value (Table 5). The improved flame retardancy of the PA6/AS/DA/25A fabrics is also reflected in the achievement of a UL-94 V-2 rating. However, TGA results of residues remaining at 600 °C in nitrogen (see Table 7) suggest that the presence of AS and DP and subsequent addition of Cloisite 25A, both promote char formation in PA6, which effects are not observed in the PA6/AlPi/25A formulations.

In terms of tensile properties, the effect of addition of 2 wt % Cloisite 25A clay to both PA6/AlPi and PA6/AS/DP formulated yarns is consistent in that respective reductions in initial Young’s modulus, tenacity and elongation-at-break values occurring as a consequence of either flame retardant present alone, are partly restored. Finally and in spite of the known thermal degradation occurring in PA6/AS/DP formulations, which have been reported to be significant at temperatures ≥ 270 °C, the acceptable filament tensile properties indicate that these effects are not greatly significant when extrusion temperatures of about 245 °C are used. This then suggests that the PA6/AS/DP/clay system described in this work could lay the basis for a possible scaling up for full commercial evaluation as a flame resistant polyamide fibre. However, one property of ammonium sulphamate requiring mention is its water solubility which could limit the wash durability of these fibres and so the impact of this would require consideration in any such evaluation.

## Figures and Tables

**Table 1:** PA6 sample matrices with AlPi, AS + DP and Cloisite 25A clay and sample types and test methods.

Formulation	PA6%	AlPi%	AS%	DP%	25A%	Sample types and test methods		
						Tensiles	LOI & UL94	Flame spread
PA6a and PA6b	100	-	-	-	-	Fil (PO, FO)	pl	fab
PA6a/25A	98.0	-	-	-	2.0	Fil (PO)	pl	fab
PA6a/AlPi	90.0	10.0	-	-	-	Fil (PO)	pl	fab
PA6a/AlPi/25A	88.0	10.0	-	-	2.0		pl	fab
PA6b/AS/DP	96.5	-	2.5	1.0	-	Fil (PO, FO)	pl	fab
PA6b/AS/DP/25A	94.5	-	2.5	1.0	2.0		pl	fab

**K**ey: pl = plaques; Fil = filament yarns; fab = fabric; PO = partly orientated or drawn; FO = fully orientated or drawn.

**Table 2:** Relative tensile properties for PA6 filament yarns.

Sample	Linear density, tex	Linear density, tex	Tenacity	Elongation-at–break
*PA6a* *^,a^	*86*	*1.00 (=110 cN/tex)*	*1.00 (=18 cN/tex)*	*1.00 (= 292%)*
PA6/25A(2%) ^a^	86	0.87	1.00	0.83
PA6/AlPi(10%) ^a^	82	0.97	0.44	0.57
PA6/AlPi(10%)/25A(2%) ^a^	87	1.35	0.78	0.83
PA 6b * ^b^	*77*	*1.0 (=47 cN/tex)*	*1.0 (=7 cN/tex)*	*1.0 (=221%)*
PA6/AS(2.5%)/DP(1%) ^b^	101	1.40	0.57	1.09
PA6/AS(2.5%)/DP(1%)/25A(2%) ^b^	101	1.76	0.71	1.36

* Control sample values in italics with actual tensile property values in brackets; Average coefficient of variation ^a^ = 11%; ^b^ = 17% of variation.

**Table 3:** Physical properties of PA6/AS/DP/Clay formulated yarns after drawing with a cold draw ratio of 2:1 (total draw ratio = 2.6:1).

Sample	Yarn linear density tex	Initial Young’s modulus cN/tex	Tenacity cN/tex	Elongation-at-break%
PA 6b	38	102 ± 14	22 ± 2.0	26 ± 4
PA6/AS(2.5%)/DP(1%)	56	153 ± 23	12 ± 1.0	27 ± 6
PA6/AS(2.5%)/DP(1%)/25A(2%)	49	176 ± 9	13 ± 1.0	33 ± 5

**Table 4:** Limiting oxygen index values of PA6 plaques containing AlPi or AS/DP and/or Cloisite 25A.

Sample	LOI (vol %)
PA6a	22.3
PA6/25A(2%)	20.1
PA6/AlPi(10%)	27.5
PA6/AlPi(10%)/25A(2%)	29.0
PA6b	20.8
PA6/AS(2.5%)/DP(1%)	27.7
PA6/AS(2.5%)/DP(1%)/25A(2%)	24.9

**Table 5:** Flame spread and dripping rates (DR) from UL94 results for PA6 plaque samples.

Sample	UL-94 Horizontal				UL-94-Vertical			
	*t_1_*, s	*t_2_*, s	Dripping rate, DR, s^−1^	Rating	*t_1_*, s	*t_2_*, s	Dripping rate, DR, s^−1^	Rating
PA6a	BC	-	1.0	HB	56	20	1.2	NR
PA6/25A(2%)	BC	-	0.7	HB	BC	-	0.9	NR
PA6/AlPi(10%)	0	-	0	HB	0	44	0.01	NR
PA6/AlPi(10%)/25A(2%)	2	36	0	HB	49	16	0.04	NR
PA6/AS(2.5%)/DP(1%)	5	2	0.3	HB	2	3	0.7	V-2
PA6/AS(2.5%)/DP(1%)/25A(2%)	4	2	0.6	HB	3	10	1.7	V-2

*t*_1_ = average burning time after the first application of flame; *t*_2_ = average burning time after the second application of flame; NR = not rated; BC = burns to clamp; HB = horizontal burn.

**Table 6:** Flame spread results for knitted PA6 fabrics comprising of AlPi and/or AS/DP and/or Cloisite 25A.

Sample	Area density of fabric, g/m^2^	Total burn time, (CV%) s	Burnt length, (CV%) mm	Flame spread rate, (CV%) mm/s	Dripping rate, DR, (CV%) s^−1^
PA6a	400	61 (27)	173 (14)	3.0 (26)	1.8 (16)
PA6/25A(2%)	505	61 (14)	BL	3.0 (8)	1.9 (12)
PA6/AlPi(10%)	412	22 (34)	173 (5)	9.4 (60)	0.3 (86)
PA6/AlPi(10%)/25A(2%)	453	52 (28)	BL	4.0 (21)	0.1 (49)
PA 6b	638	51 (9)	131 (9)	2.6 (22)	2.2 (12)
PA6/AS(2.5%)/DP(1%)	542	31 (20)	51 (17)	1.6 (34)	1.4 (16)
PA6/AS(2.5%)/DP(1%)/25A(2%)	578	23 (19)	40 (25)	1.7 (24)	1.0 (18)

BL indicates sample burnt entire length, i.e., ≥ 180 mm; CV = coefficient of variation.

**Table 7:** TGA mass loss temperatures and residual chars under air and nitrogen of PA6 containing AlPi and/or Cloisite 25A; Values in the parentheses are in nitrogen.

Samples	*T*_5%_, °C	*T*_10%_, °C	*T*_50%_, °C	Residue at 600 °C, wt %
PA6a	364 (388)	391 (407)	436 (444)	0.0 (1.1)
PA6/25A(2%)	355 (374)	388 (400)	436 (440)	1.8 (1.4)
PA6/AlPi(10%)	374 (379)	398 (398)	434 (432)	4.6 (2.0)
PA6/AlPi(10%)/25A(2%)	361 (368)	396 (394)	435 (430)	5.1 (2.4)
PA6b	371 (382)	391 (406)	437 (446)	0.0 (0.3)
PA6/AS(2.5%)/DP(1%)	344 (352)	389 (395)	442 (443)	0.2 (1.4)
PA6/AS(2.5%)/DP(1%)/25A(2%)	295 (331)	366 (387)	435 (439)	2.0 (4.2)
